# ST-CellSeg: Cell segmentation for imaging-based spatial transcriptomics using multi-scale manifold learning

**DOI:** 10.1371/journal.pcbi.1012254

**Published:** 2024-06-27

**Authors:** Youcheng Li, Leann Lac, Qian Liu, Pingzhao Hu

**Affiliations:** 1 Department of Biochemistry, Schulich School of Medicine & Dentistry, Western University, London, Ontario, Canada; 2 Department of Computer Science, Western University, London, Ontario, Canada; 3 Department of Computer Science, University of Manitoba, Winnipeg, Manitoba, Canada; 4 Department of Statistics, University of Manitoba, Winnipeg, Manitoba, Canada; 5 Department of Applied Computer Science, University of Winnipeg, Winnipeg, Manitoba, Canada; 6 Department of Epidemiology and Biostatistics, Schulich School of Medicine & Dentistry, Western University, London, Ontario, Canada; 7 Department of Oncology, Schulich School of Medicine & Dentistry, Western University, London, Ontario, Canada; 8 The Children’s Health Research Institute, Lawson Health Research Institute, London, Ontario, Canada; Carnegie Mellon University, UNITED STATES

## Abstract

Spatial transcriptomics has gained popularity over the past decade due to its ability to evaluate transcriptome data while preserving spatial information. Cell segmentation is a crucial step in spatial transcriptomic analysis, as it enables the avoidance of unpredictable tissue disentanglement steps. Although high-quality cell segmentation algorithms can aid in the extraction of valuable data, traditional methods are frequently non-spatial, do not account for spatial information efficiently, and perform poorly when confronted with the problem of spatial transcriptome cell segmentation with varying shapes. In this study, we propose ST-CellSeg, an image-based machine learning method for spatial transcriptomics that uses manifold for cell segmentation and is novel in its consideration of multi-scale information. We first construct a fully connected graph which acts as a spatial transcriptomic manifold. Using multi-scale data, we then determine the low-dimensional spatial probability distribution representation for cell segmentation. Using the adjusted Rand index (ARI), normalized mutual information (NMI), and Silhouette coefficient (SC) as model performance measures, the proposed algorithm significantly outperforms baseline models in selected datasets and is efficient in computational complexity.

## Author summary

Spatial transcriptomics data is a type of biological data that describes gene expression patterns in the context of tissue or cell spatial arrangement. Traditional transcriptomics studies the gene expression of a group of cells or a tissue sample as a whole, revealing which genes are active or inactive in that sample. Spatial transcriptomics, on the other hand, is a recent technology that can maintain the spatial information of where these genes are expressed inside the tissue. These methods provide a more accurate description of tissue and cell subcellular architecture, allowing for a better understanding of physical and biochemical interactions between cells. Precise cell identification is critical because it can aid in the discovery of unusual cell types, particularly in cancer research. Traditional clustering approaches, on the other hand, frequently fail to account for spatial information. The issue in bioinformatics is thus to diversify cell segmentation approaches in spatial transcriptomic analysis. To that purpose, we develop a cell segmentation technique for spatial transcriptomic data that uses distance metrics to better define the spatial transcriptomics distribution. The experimental results reveal that this algorithm outperforms the popular cell segmentation algorithms and performs faster under the same conditions.

## 1. Introduction

Human and animal tissues consist of diverse cell types that are organized systematically [1]. Single-cell transcriptomics approaches have exploded in popularity over the past decade, and single-cell RNA sequencing (scRNA-seq) technologies have become the tool of choice for characterizing complicated tissue states [2–4]. However, these methods are resulting in a loss in spatial information. Thus, single cell sequencing methods are gradually improved by spatial transcriptomics, a recent technical invention that evaluates transcriptome information while conserving spatial information [1]. In these techniques, the transcriptome measurements are resolved based on situ sequencing, multi-channel single-molecule fluorescent in situ hybridization (smFISH) [5–7], or spatial barcode hybridization [8, 9].

Furthermore, the effects of physical and biochemical interactions between cells, as well as the effects of transcriptomic processes on tissue organization during development and disease [10, 11], can be revealed. The number of genes and molecules that can be detected in most assays is currently limited to 30 to 300 genes and 50 to 500 molecules per cell, respectively [12]. Increasing scale and spatial resolution has enabled an accurate description of the subcellular organization of tissue and cells [13, 14], as the number of genes optimized for detection has increased to thousands [15]. Thus, spatial transcriptomics analysis may eventually replace scRNA-seq since they provide technical benefits such as the ability to avoid the capricious tissue disentanglement steps required by scRNA-Seq.

The most fundamental task in spatial transcriptomics data analysis is cell type identification [16]. The process of identifying the cell type of each spatial unit or spot typically starts with dimensionality reduction techniques to reduce the temporal and spatial complexity of downstream analysis. Cells are clustered using the simplified representation under the assumption that cells of the same type belong to the same cluster [1]. Cell segmentation is a critical step in spatial transcriptomic analysis. High-quality cell segmentation algorithms can assist people in mining valuable data. Cells have a variety of irregular shapes; however, traditional clustering methods are frequently non-spatial and do not efficiently account for spatial information [17]. As a result, because cell deformation and cell overlap undermine the spatial assumption, these methods fail to perform well in the problem of spatial transcriptome cell segmentation with different shapes. Several state-of-the-art methods have been proposed for cell segmentation. ClusterMAP [18] is proposed as an annotation-free unsupervised clustering framework for spatial gene expression clustering using neighborhood gene composition. SpaGCN [19] uses graph convolutional network (GCN) and considers the similarity between adjacent spots to account for gene expression spatial dependency. A graph attention auto-encoder framework [17] is developed to characterize the spatial similarity at spatial domain boundaries. Moreover, Cellpose [20] is considered as a state-of-art cell segmentation algorithm on variety of image types. In spatial transcriptome analysis, a manually designed distance metric is useful to describe the relationship between spatial transcriptome sampling points. Thus, in this study, we develop ST-CellSeg, a cell segmentation algorithm for spatial transcriptomics by employing the concept of manifold to better describe the spatial transcriptome distribution under this distance measure. A manifold is a space with local Euclidean space properties that is used to describe geometric shapes in mathematics. The proposed algorithm is a three-stage algorithm. The first stage is to construct a fully connected graph and learn its manifold structure. The second stage is to find a low-dimensional spatial probability distribution representation that approximates the high-dimensional manifold structure. Assuming the location of each transcribed RNA has a strong relationship to its neighbors; the novelty of our method is to use a multi-scale neighborhood gene composition (MSNGC) feature to represent the spatial information of the spatial transcriptome. The advance of using MSNGC in compared to single scale neighbor gene composition is that MSNGC can gather more information of cells. The designed distance representation is then used to fuse spatial coordinate information and multi-scale neighborhood gene composition feature information. In final stage, considering the distribution between each cluster, we use density clustering method to segment cells in low-dimensional space and feed the loss of density clustering back to the upstream training process. To assess the performance of our proposed algorithm, we apply it to various datasets and compare the performance to state-of-the-art spatial transcriptomic cell segmentation algorithms. Using the cluster analysis index and the number of floating points as evaluation metrics, the experimental results show that our algorithm outperforms other baseline methods on the cluster analysis index and has a faster speed under the same conditions.

## 2. Materials and methods

### 2.1 Datasets

To evaluate the performance of the proposed method, we consider three spatial transcriptomic datasets with different gene distribution: STARmap mouse placenta 903-gene [15], STARmap cardiac organoid 8-gene [21], MERFISH mouse POA [14]. In this study, we rename these datasets as *STARmap 903-gene data*, *STARmap 8-gene data*, and *MERFISH 140-gene data*, respectively. These data were collected from a variety of experiments in which different gene expression shapes cell types onto a three-dimensional (3D) space using three image-based in situ transcriptomics methods: spatially resolved transcript amplicon readout mapping (STARmap) [15] and multiplexed error-robust fluorescence in situ hybridization (MERFISH) [14]. **Table 1** provides a description of these datasets, including experiment methods, number of genes, number of cells, number of reads, and cell types.

**Table 1:** Summary of spatial transcriptome datasets.

Datasets	Experimental methods	Reads	Genes	Cells	Cell types
STARmap 903-gene data	STARmap	5,090,930	903	7,224	12
STARmap 8-gene data	STARmap	47,594	8	1,519	3
MERFISH 140-gene data	MERFISH	3,065,171	140	10,320	9

The basic unit of spatial transcriptomic data is spot. As indicated in **Table 1**, reads represent the number of spots in each dataset.

### 2.2 Data Preprocessing

Before performing data dimensionality reduction, it is essential to preprocess the data to ensure its quality and remove any outliers that may negatively impact subsequent analysis. In this step, we calculate the distance between each data point and its nearest neighbor. If the distance exceeds a predefined threshold, the point is considered an outlier and filtered out from further analysis. This approach helps remove data points that might introduce noise or bias to the clustering results, ensuring a more reliable and accurate outcome.

### 2.3 Spatial transcriptomic uniform manifold approximation

**Fig 1:**
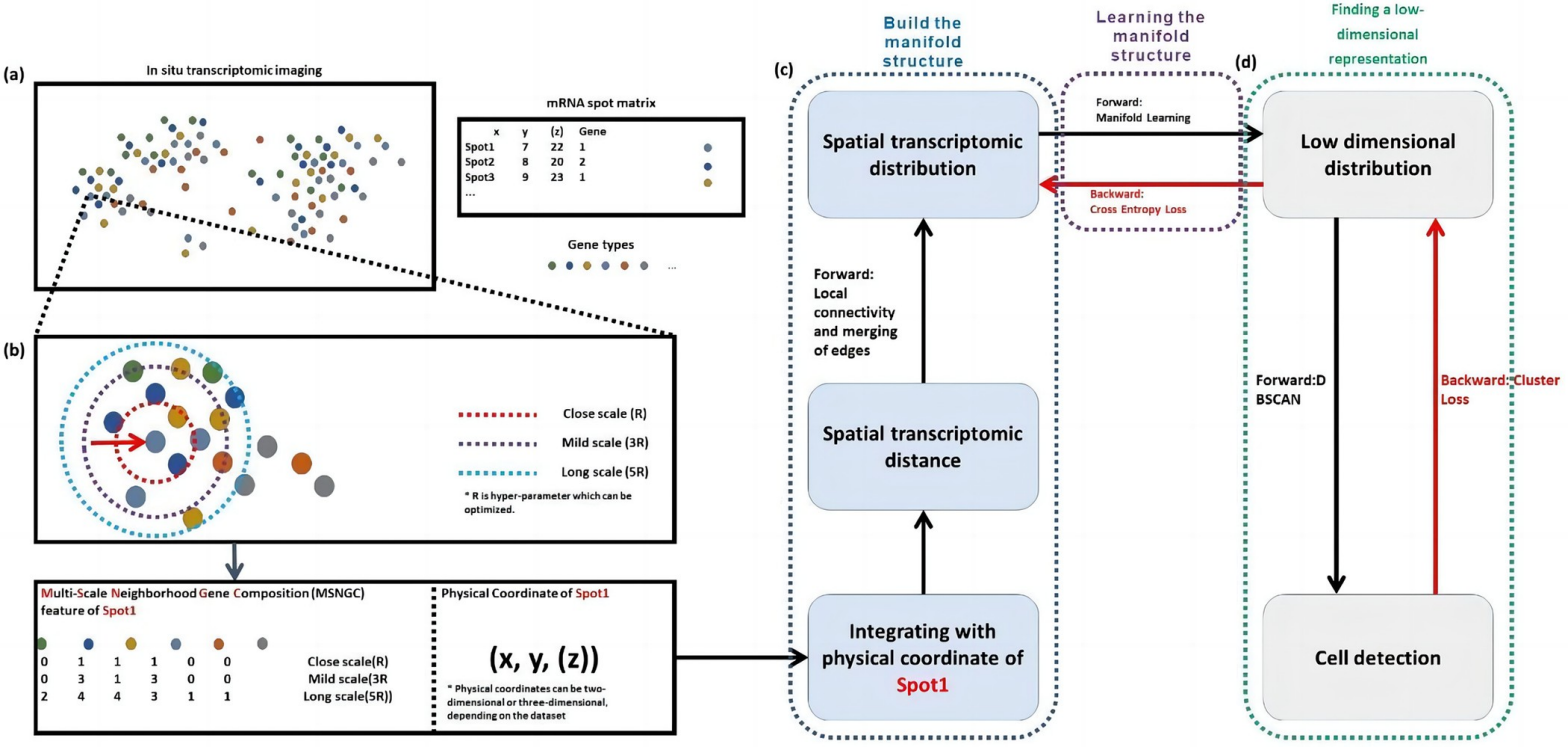
The diagram of ST-CellSeg for cell segmentation. The ST-CellSeg cell segmentation algorithm has three stages. (a) A spatial transcriptomic data structure. Each spatial transcriptome sampling site includes a gene tag and physical coordinates, which can be two-dimensional or three-dimensional. (b) Illustration of multi-scale neighbourhood gene composition (MSNGC). MSNGC is a spot feature that counts the number of adjacent genes at different scale levels and represents the gene relationship between the sampling point and its surroundings. Given MSNGC features and physical coordinates of spots, ST distances are calculated, which represent the spatial location of sampling points. (c-d) An ST-CellSeg cell segmentation framework. Spatial transcriptomic manifolds are built in (c). Local connectivity and merging edges are combined to build a manifold structure. The low dimensional clustering space is constructed from manifold learning. (d) Cell segmentation (or cell detection) results are obtained from density clustering in the clustering space. Fusion cross-entropy and clustering analysis metrics are used as error propagation.

In this study, we propose a spatial transcriptomic uniform manifold approximation algorithm for cell segmentation (ST-CellSeg) by taking multi-scale information into account. ST-CellSeg is an extension of uniform manifold approximation (UMAP) [22] learning algorithm for cell segmentation in spatial transcriptome. The proposed algorithm maps to the space for clustering of segmented cells by learning the manifold structure of the spatial transcriptome data. The entire cell segmentation using ST-CellSeg includes three steps. The first step is to learn the manifold structure of a fully connected graph which is constructed based on multi-scale distance metric of the spatial transcriptome. The second step is to find a low-dimensional spatial probability distribution representation that approximates the high-dimensional manifold structure. Finally, given the structure of manifold is learned in Euclidean space, cell segmentation is conducted based on the density clustering method (i.e., sample points are clustered in low-dimensional space). The overview of ST-CellSeg architecture is described in **Fig 1**.

#### 2.3.1 Data structure of spatial transcriptomic data and basic notations

Spatial transcriptomic data is a set of disordered points or *spots*. Each spot has two primary components: physical coordinates and a gene tag. While the physical coordinates satisfy the properties of symmetry, positive qualitative, and triangular inequalities, the gene tags represent the gene categories corresponding to the sampling points in discrete disorder. The ST sampling points are generally assumed to have local correlation which is the basis of the spatial transcriptome distribution. To better understand the structure of spatial transcriptomic data, let $S_{i}$ be the *i*^*th*^ spot which is the basic unit of the spatial transcriptome dataset, for *i* = 1,2,…*N* where *N* is the total number of spots. The number of spots in each ST datasets is defined as reads in **Table 1**. We can define each spot $S_{i}$ as

$$S_{i}≐(x,y,(z)),$$ (1)

where $S_{i}$ is the two-dimensional or three-dimensional physical coordinate of each spot. Each $S_{i}$ is attached to a gene tag ℓ for ℓ = 1,2,…*L*. An example of ST data structure is illustrated in **Fig 1, Panel (a)**.

#### 2.3.2 Spatial transcriptome distance

To compute the spatial transcriptome distance of spots $S_{i}$ and $S_{j}$, denoted as $D(S_{i},S_{j})$, we propose the multi-scale neighborhood gene composition (MSNGC) features of each spot. MSNGC feature represents the gene relationship of a given spot $S_{i}$ and its surrounding sampling points. Denote **M**_*i*_ a *c*×*L* MSNGC feature matrix of spot $S_{i}$ where *c* represents the number of scale levels and *L* indicates the number of gene tags. We consider three different scales: close scale (*R*), mid scale (3*R*), and long scale (5*R*) where *R* is the radius of circular surrounding given spot $S_{i}$. Each element of **M**_*i*_ represent the count of surrounding sampling points of $S_{i}$ having gene tag and scale level as indicating by column and row labels of the matrix. The illustration can be found in **Fig 1, Panel (b).** Let **M**_*i*_ and **M**_*j*_ be MSNGC features of spots $S_{i}$ and $S_{j}$, respectively. We propose to build a manifold using multiscale information by considering information from all three scales (*R*, 3*R*, and 5*R*), and the Pearson correlation factors of **M**_*i*_ and **M**_*j*_ can be defined as

$$\rho_{\mathrm{MSNGC}}(M_{i},M_{j})=\sum_{\beta =1}^{3}\frac{\sum_{l=1}^{L}\left[{\left(M_{i}\right)}_{l\beta }-{\bar{\left(M_{i}\right)}}_{\beta }\right]\times [{\left(M_{j}\right)}_{l\beta }-{\bar{\left(M_{j}\right)}}_{\beta }]}{\sqrt{\sum_{l=1}^{L}{\left[{\left(M_{i}\right)}_{l\beta }-{\bar{\left(M_{i}\right)}}_{\beta }\right]}^{2}\sum_{l=1}^{L}{\left[{\left(M_{j}\right)}_{l\beta }-{\bar{\left(M_{j}\right)}}_{\beta }\right]}^{2}}},$$ (2)

where *β* represents three different scales, ${\bar{\left(M_{i}\right)}}_{\beta }$ and ${\bar{\left(M_{j}\right)}}_{\beta }$ are the average of MSNGC features of spots $S_{i}$ and $S_{j}$ in scale *β*, respectively.

Given *ρ*_*MSNGC*_(**M**_*i*_, **M**_*j*_), the ST distance $D(S_{i},S_{j})$ of any given spots $S_{i}$ and $S_{j}$ can expressed as

$$D(S_{i},S_{j})=d(S_{i},S_{j})\times \left(1-\rho_{MSNGC}(M_{i},M_{j})\right),$$ (3)

where $d(S_{i},S_{j})$ represents the Manhattan distance of spots $S_{i}$ and $S_{j}$. Although the distances may not satisfy the properties of triangle inequality in some cases, ST-CellSeg only requires the distances satisfying the properties of symmetry and non-negativity. Thus, the performance of the proposed method does not depend on the triangle inequality of the distances. Furthermore, among the commonly used distance measures, Euclidean distance and Manhattan distance are two most popular ones in clustering data mining techniques [23]. Manhattan distance has been used in clustering algorithms for better performance [24, 25]. Apart from this reason, we have decided to choose Manhattan distance over Euclidean distance in our proposed method because of its primary advantage in computational efficiency and cost. The process of computing the ST distance is describe in **Fig 1, Panels (b) and (c).**

#### 2.3.3 Spatial transcriptomic manifold in high-dimensional space

To determine the ST manifold in high-dimensional space, a weighted k-neighbour graph is constructed. Given the preferred number of neighbours k as an input hyper-parameter, we consider the k-nearest neighbour descent (k NN-descent) algorithm to find the nearest neighbor spots. The k NN-descent forms a wider connection across manifold with larger k, while the algorithm pays more attention to local information with smaller k. We assume the sample spots are evenly distributed across the manifold and distance measure is considered varied between regions. Denote $S=(S_{1},S_{2},\ldots ,S_{N})$ be the input set of spots and let $K=\{S_{i,1},S_{i,2},\ldots ,S_{i,k}\}$ be the set of k nearest spots of spot $S_{i}$. We define a weighted (directed) graph G=(V,E,w) where vertices **V** of G is the set S and edges **E** is simply the set K. The edge weight $w(S_{i},S_{i,j})$ between two $S_{i}$ and its neighbours can be expressed as

$$w(S_{i},S_{i,j})=\exp(\frac{-\max\{0,D(S_{i},S_{i,j})-\delta_{i}\}}{\sigma_{i}}),$$ (4)

where $\delta_{i}=\min\{D(S_{i},S_{i,j})|1\leq j\leq k,1\leq i\leq N\}$ is the distance to the nearest neighbor of $S_{i}$, and the normalizing factor *σ*_*i*_ is set in condition to

$$\sum_{j=1}^{k}\exp\left(\frac{-\max\{0,D(S_{i},S_{i,j})-\delta_{i}\}}{\sigma_{i}}\right)=\log_{2}(k).$$ (5)

Although the edge weight of spot $S_{i}$ to $S_{j}$ is different from that of $S_{j}$ to $S_{i}$, ST-CellSeg overcomes this inconsistency by taking the union of two edges and constructs the related weighted (undirected) graph $G^{\prime }$ by connecting k nearest neighbours. The weighted adjacency matrix A of $G^{\prime }$ is defined as

$$A_{ij}=w(S_{i},S_{j})+w(S_{j},S_{i})-w(S_{i},S_{j})w(S_{j},S_{i}),\;\mathrm{for}\;i,j=1,2,\ldots N.$$ (6)

In the high-dimensional probability distribution for modeling ST-CellSeg, a Bayesian distribution between two spots $S_{i}$ and $S_{j}$ is considered. Based on the smooth nearest neighbour distances, the similarities *p*_*j*|*i*_ is defined as

$$p_{j|i}=\exp(-\frac{D(S_{i},S_{i,j})-\delta_{i}}{\sigma_{i}}),$$ (7)

and the symmetrization can be expressed as $p_{ij}=p_{i|j}+p_{j|i}-p_{i|j}p_{j|i}$.

In the context of spatial transcriptomic analysis, the purpose of probability symmetrization is to refine the estimation of spatial relationships between transcriptomics spots. In high-dimensional distributions where each spot represents a complex set of transcriptomic data, defining a reliable measure of similarity between spots is important for accurate segmentation and cell type identification.

The similarity metric is initially derived using Bayesian distribution to account for the inherent uncertainty and variability in the spatial data. This similarity is based on the smooth nearest neighbour distances, which incorporate both the proximity and the density of spots to capture the local structure of the data more effectively. By considering Bayesian probabilities, the model can infer the degree to which spots share similar transcriptomic profiles, which might suggest a functional or cellular relationship.

The symmetrization of the probability is used for two reasons. First, it ensures that the similarity measure is bidirectional and reflects the mutual relationship between two spots irrespective of the order in which they are considered. This is crucial in spatial analysis, where the interaction between two locations is inherently reciprocal. Symmetrization can be used to prevent the model from overemphasizing unidirectional similarities that could arise from asymmetries in the local density of spots or from noise in the data.

Secondly, symmetrization promotes a more robust clustering of spots, as it balances the similarity contributions from both spots. For example, in the presence of outliers or spots with disproportionately high or low density of transcripts, the raw nearest neighbour distances might skew the similarity metric, affecting the downstream clustering or segmentation. By averaging the contributions from each spot, the symmetrization reduces the influence of such anomalies, leading to more stable and interpretable clusters that are more reflective of the underlying cellular architecture.

#### 2.3.4 Spatial transcriptomic manifold in clustering space

Given the approximate manifold learned from a higher-dimensional space in previous subsection, we now obtain the ST manifold in clustering space which is a lower-dimensional representation prior to conduct the cell segmentation. However, the distance is variable in low-dimensional space. To construct a spatial transcriptomic manifold in cluster space, we consider the standard Euclidean distance relative to the global coordinate system. The low-dimensional similarities *q*_*ij*_ are defined as

$$q_{ij}={\left(1+a∥S_{i}^{\prime }-S_{j}^{\prime }∥\begin{array}{c} 2b\\ 2 \end{array}\right)}^{-1},$$ (8)

where *a*, *b* are defined positive-valued hyperparameters, and $S_{i}^{\prime }$ and $S_{j}^{\prime }$ are representation of $S_{i}$ and $S_{j}$ in clustering space.

Since the conversion from variable distance to standard distance may affect the distance to the nearest neighbor, a hyperparameter *d*_*min*_ defining the minimum distance between points in lower dimensional space is required. Given *d*_*min*_, the algorithm searches for a better representation to replace the representation of low-dimensional manifold. ST-CellSeg is implemented by minimizing the cross entropy (CE) cost function which is defined as

$$CE_{ST-UMAP}=\sum_{i\neq j}[p_{ij}\log\frac{p_{ij}}{q_{ij}}+(1-p_{ij})\log\frac{1-p_{ij}}{1-q_{ij}}]$$ (9)

The lower-dimensional representation is then used for clustering. This step is shown in **Fig 1, Panels (c) and (d)**. The multi-scale neighborhood gene composition (MSNGC) manifold learning of ST-CellSeg is presented in **Algorithm 1**.

**Algorithm 1.** ST-CellSeg algorithm in MSNGC manifold learning

1: **input** Spots S, number of nearest neighbours k, number of epochs *e*,

2: clustering space dimension *d*

3: **initialize**

4: Obtain *N*, the total number of spots $S_{i}$ for *i* = 1,2,…,*N*.

5: **for** *i*,*j* = 1,2,…,*N* **do**

6: Compute **M**_*i*_ and **M**_*j*_.

7: Calculate the spatial transcriptome distance $D(S_{i},S_{j})$ using Eq 3.

8: Obtain edge weight $w(S_{i},S_{i,j})$ using Eq 4.

9: Construct $A_{ij}$ from Eq 6.

10: Obtain degree matrix **A**^*D*^ of graph A.

11: Compute $L=\sqrt{A^{D}}(A^{D}-A)\sqrt{A^{D}}$.

12: Obtain **v** where **v** is the sorted eigenvectors of **L**.

13: Assign $S^{\prime }\leftarrow v[1\ldots d+1]$

14: **for** *t* = 1,2,…,*e* **do**

15: Optimize embedding by minimizing (9).

16: Compute ${C=DBSCAN(S}^{\prime })$

17: Obtain center spots $S_{i}$ for *i* = 1,2,…,card(C) of each cluster C

18: **for** *i*,*j* = 1,2,…,card(C) **do**

19:   Compute **M**_*i*_ and **M**_*j*_.

20:   Calculate the spatial transcriptome distance $D(S_{i},S_{j})$ using Equation

21:   Obtain edge weight $w(S_{i},S_{i,j})$ using Eq 4.

22:   Add $A_{ij}$ from Eq 6.

23:   Obtain degree matrix **A**^*D*^ of graph A.

24:   Compute $L=\sqrt{A^{D}}(A^{D}-A)\sqrt{A^{D}}$.

25:   Obtain **v** where **v** is the sorted eigenvectors of **L**.

26:   Assign $S^{\prime }\leftarrow v[1\ldots d+1]$

27: **output** Lower-dimensional representation $S^{\prime }$.

#### 2.3.5 Cell segmentation of spatial transcriptomic data

**Fig 2:**
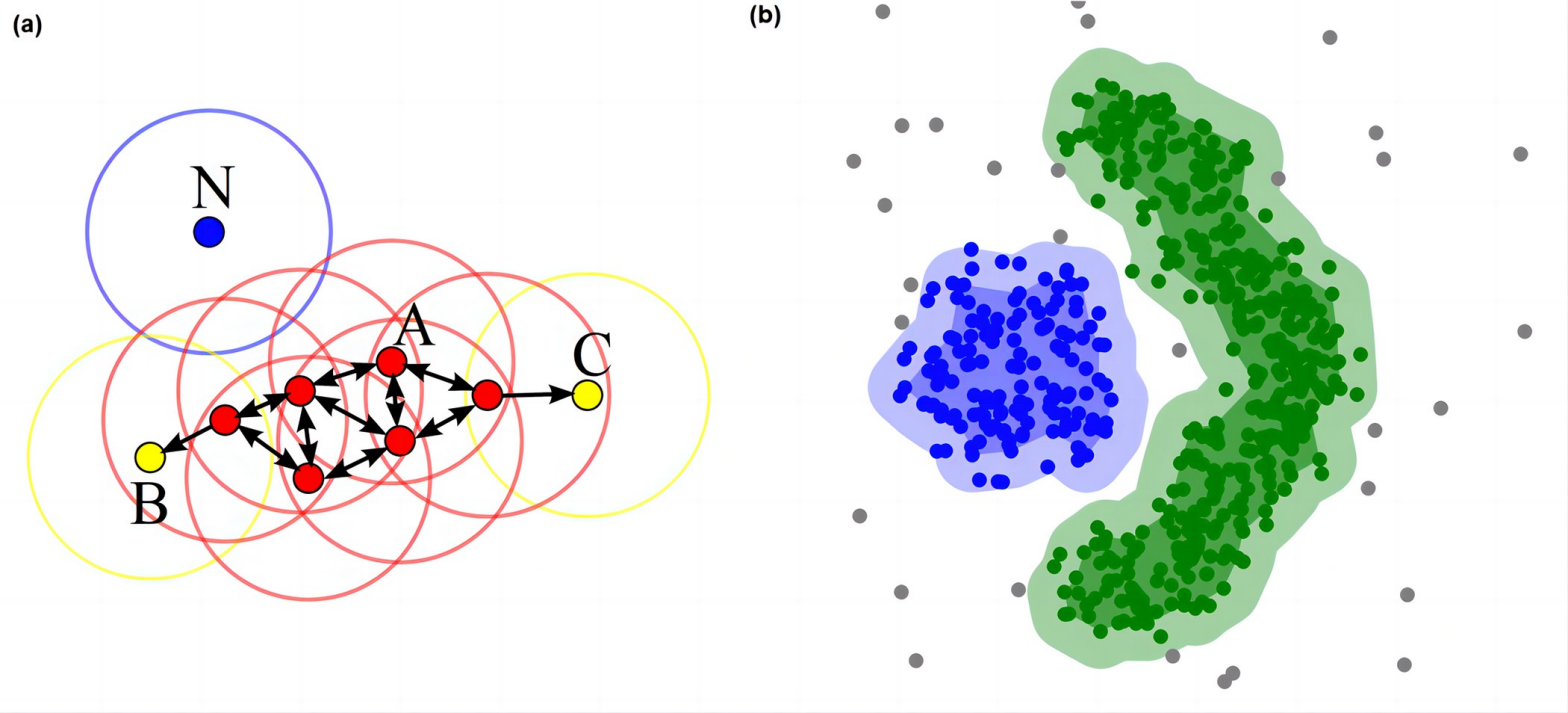
Cell segmentation with DBSCAN. (a) DBSCAN Workflow: Core Points, Reachability, and Noise. In the DBSCAN workflow with minPts = 4, core points (including Point A) are identified by the condition that the area surrounding them within an ε radius contains at least 4 points. These core points form a single cluster as they are mutually reachable. Points B and C, although not core points, are reachable from Point A through other core points, thus belonging to the same cluster. Point N is classified as a noise point since it is neither a core point nor directly reachable from any core points. (b) Illustration of clustering effect using DBSCAN.

Given the manifold in lower dimension, we conduct the cell segmentation using density-based spatial clustering of applications with noise (DBSCAN) algorithm in Euclidean space. DBSCAN can partition regions with sufficiently high density into clusters and to find clusters of arbitrary shapes in noisy spatial data. The overview of cell segmentation using DBSCAN is summarized in **Fig 2**.

The DBSCAN algorithm requires a scan radius (*eps*) and a minimum number of included points (*minPts*) as inputs. The algorithm starts by choosing any unvisited point to visit and scans all nearby points which are within the distance of *eps* (including *eps*). If the number of nearby points is greater or equal to *minPts*, the current point forms a cluster with its nearby points, and the starting point is marked as visited. The algorithm will continuously process all points in the cluster that are not marked as visited in a recursive manner to extend the cluster. However, if the number of nearby points is smaller than *minPts*, the point is temporarily marked as a noise point. If the cluster is sufficiently expanded (i.e., all points in the cluster are marked as visited), the same algorithm is applied to the unvisited points. Given several clusters obtained from DBSCAN, we select several sample points closest to the centre of each cluster. In addition, distribution of relationship between obtained clusters in the manifold learning process can be learned.

### 2.4 Hyper-parameter settings

In the ST-CellSeg framework, the values of hyper-parameters may have impact on the performance of the algorithm in cell segmentation. The optimal values of the number of neighbors k, learning rate *α*, number of epochs *e*, radius to search for circles to build ST manifold *r*, *eps* and *minsamples* in DBSCAN are reported in **Table 2**.

**Table 2:** Optimal values of hyper-parameters used in ST-CellSeg framework.

Hyper-parameters	Optimal values
k	Between 2 and 100, and the optimal value is **50.**
*α*	The initial learning rate value is 1.0
*e*	200 for large datasets; 500 for small datasets
*r*	10 in *x*, *y* domain; 7 in *z* domain
*eps*	Hyperparameter in DBSCAN which is a maximum distance from one observation to another before they are no longer considered as neighbors
minsamples	Default value is 1.

### 2.5 Baseline methods

**ClusterMAP** [18]: ClusterMAP is an annotation-free unsupervised clustering framework for multi-scale spatial gene expression clustering. The algorithm can be used to precisely cluster RNAs into subcellular structures in both two- and three-dimensional space, incorporating the physical location and gene identity from images with high-dimensional transcriptomic profiles.

**SpaGCN** [19]: SpaGCN is a novel clustering approach that incorporates spatial information to account for the spatial dependency of gene expression. This method primarily analyzes gene expression count matrices, emphasizing the relationships between adjacent spots. SpaGCN uses a graph convolutional network (GCN) to effectively integrate gene expression data with the spatial locations of the spots. Additionally, it employs a self-supervised learning module to discover spatial domains within the tissue.

**Baysor** [26]: Baysor is a cell segmentation method for spatial transcriptomics data. It optimizes cell boundaries by considering the joint likelihood of transcriptional composition and cell morphology. The algorithm integrates gene expression and spatial location, utilizing a self-supervised module to identify domains.

**STAGATE** [17]: STAGATE is a graph attention auto-encoder framework used to characterize spatial similarity at spatial domain boundaries. By integrating spatial information and pre-clustering of gene expression profiles, the similarity of neighboring spots is learned in low-dimensional latent embedding and a cell type-aware module.

**Cellpose** [20]: Cellpose is an interface to state-of-art nuclei segmentation algorithm that can perform cell segmentation on a variety of image types. The model does not require model retraining or parameter adjustments. This generalized machine learning segmentation method can also reuse the two-dimensional (2D) model for three-dimensional (3D) extension without using 3D-labeled data.

**StarDist** [27]: StarDist is a cell detection method that predicts a shape representation without requiring any refinement. The StarDist algorithm employs a thin neural network based on U-Net [28]. Although the algorithm is simple to learn and apply, the localization accuracy can compete with other cutting-edge methods.

### 2.6 Performance metrics

To evaluate the performance in cell segmentation of ST-CellSeg, we consider three evaluation metrics: adjusted Rand index (ARI), normalized mutual information (NMI), and Silhouette coefficient (SC). In addition, floating point operations per second (FLOPs) is used as a measure to evaluate the time complexity of the proposed algorithm.

**Adjusted Rand index (ARI):** ARI is the variant of Rand index metric. This metric is used to generally evaluate the similarity between two clusters. The score of ARI is between 0 and 1 where 0 represents a random result, 1 represents a complete agreement between the clusters, and negatives value indicates the index is smaller than the expected one [29]. The equation for calculating the ARI can be defined as

$$ARI=\frac{\sum_{ij}\left(\begin{array}{r} n_{ij}\\ 2 \end{array}\right)-[\sum_{i}\left(\begin{array}{r} n_{i}\\ 2 \end{array}\right)\sum_{j}\left(\begin{array}{r} n_{j}\\ 2 \end{array}\right)]/(\begin{array}{c} n\\ 2 \end{array})}{\frac{1}{2}[\sum_{i}\left(\begin{array}{r} n_{i}\\ 2 \end{array}\right)+\sum_{j}\left(\begin{array}{r} n_{j}\\ 2 \end{array}\right)]-[\sum_{i}\left(\begin{array}{r} n_{i}\\ 2 \end{array}\right)\sum_{j}\left(\begin{array}{r} n_{j}\\ 2 \end{array}\right)]/(\begin{array}{c} n\\ 2 \end{array})},$$ (10)

where *n*_*i*_ indicates the number of cells assigned to the *i*^*th*^ cluster, *n*_*j*_ indicates the number of cells with true label *j*, and *n*_*ij*_ indicates the size of cells having true label *i* and assigned to *j*^*th*^ cluster.

**Normalized mutual information (NMI):** NMI is the normalization of mutual information (MI) score [30] which is used to measure the similarity and exploit the grouping property. NMI normalizes MI by generalizing the means of true labels and predicted labels. The formula of NMI is defined as

$$NMI(Y,C)=\frac{2MI(Y,C)}{\left[H(Y)+H(C)\right]},$$ (11)

where *Y* is the predicted cell labels, *C* is the ground truth cell labels, *H*(*Y*) and *H*(*C*) are the entropy of true and predicted cell labels, respectively. The MI (*Y*,*C*) = *H*(*Y*)−*H*(*Y*|*C*). Thus, the value domain of NMI is [0,1], higher NMI score indicates the predicted labels are similar to the ground truth.

**Silhouette coefficient (SC):** SC is commonly used as a performance metric to evaluate performance of clustering algorithms. This coefficient measures the degree of separation between clusters by calculating the tightness and separation between clusters [31]. The score is between −1 and 1 where larger value indicates a higher degree of separation among clusters. The formula to obtain SC is defined as

$${SC}_{i}=\frac{{(b}_{i}-a_{i})}{max(a_{i},b_{i})},$$ (12)

where *a* is dissimilarity within the *i*^*th*^ cluster and *b* is the dissimilarity between the *i*^*th*^ cluster and its nearest cluster.

#### Time complexity analysis

We use both the floating-point operations per second (FLOPs) and actual time spent on the analysis by each of the algorithms. FLOPs indicating the number of floating operations per second are used to measure the complexity of model computation. This metric can be an indirect measure to calculate the speed of neural network model. The FLOPs_+_ takes value of 1 if the method is a floating-point operation, and 0 otherwise.

## 3. Results

We verify our method on three datasets and compare its performance in cell segmentation with six mainstream spatial transcriptome segmentation algorithms. To evaluate the effectiveness of our method, we conduct a series of ablation experiments to discuss the effect of different clustering methods using different scaling coefficients. Moreover, we count the number of computations of our method and the selected baseline methods to measure the time complexity of the algorithm.

### 3.1. Cell segmentation performance

We compare the differences between ST-CellSeg and six spatial transcriptome cell segmentation algorithms such as ClusterMap, SpaGCN, STAGATE, Baysor, CellPose, and StarDist on three datasets: STARmap 903-gene, STARmap 8-gene, and MERFISH 140-gene. The experimental results are shown in **Table 3**. The results show that the proposed method performs better than the six selected algorithms in all three cluster analysis metrics ARI, NMI, and SC for both STARmap 8-gene and MERFISH 140-gene data sets. ClusterMap shows the best performance for STARmap 903-gene data set when ARI metric is used. This shows that our ST-CellSeg is effective in cell segmentation.

**Table 3:** Cell segmentation performance of ST-CellSeg in comparison to six baseline models.

Datasets	STARmap 903-gene			STARmap 8-gene			MERFISH 140-gene		
Methods/Metric	ARI	NMI	SC	ARI	NMI	SC	ARI	NMI	SC
ST-CellSeg	0.84	0.96	0.86	0.85	0.95	0.90	0.81	0.91	0.91
ClusterMap	0.86	0.85	0.85	0.83	0.93	0.86	0.81	0.90	0.90
SpaGCN	0.73	0.81	0.83	0.71	0.84	0.80	0.74	0.80	0.80
STAGATE	0.78	0.80	0.80	0.77	0.84	0.78	0.77	0.80	0.78
Baysor	0.75	0.77	0.80	0.80	0.82	0.83	0.74	0.84	0.76
Cellpose	0.69	0.75	0.69	0.61	0.68	0.70	0.69	0.68	0.65
StarDist	0.67	0.63	0.70	0.59	0.71	0.74	0.64	0.63	0.66

**Fig 3:**
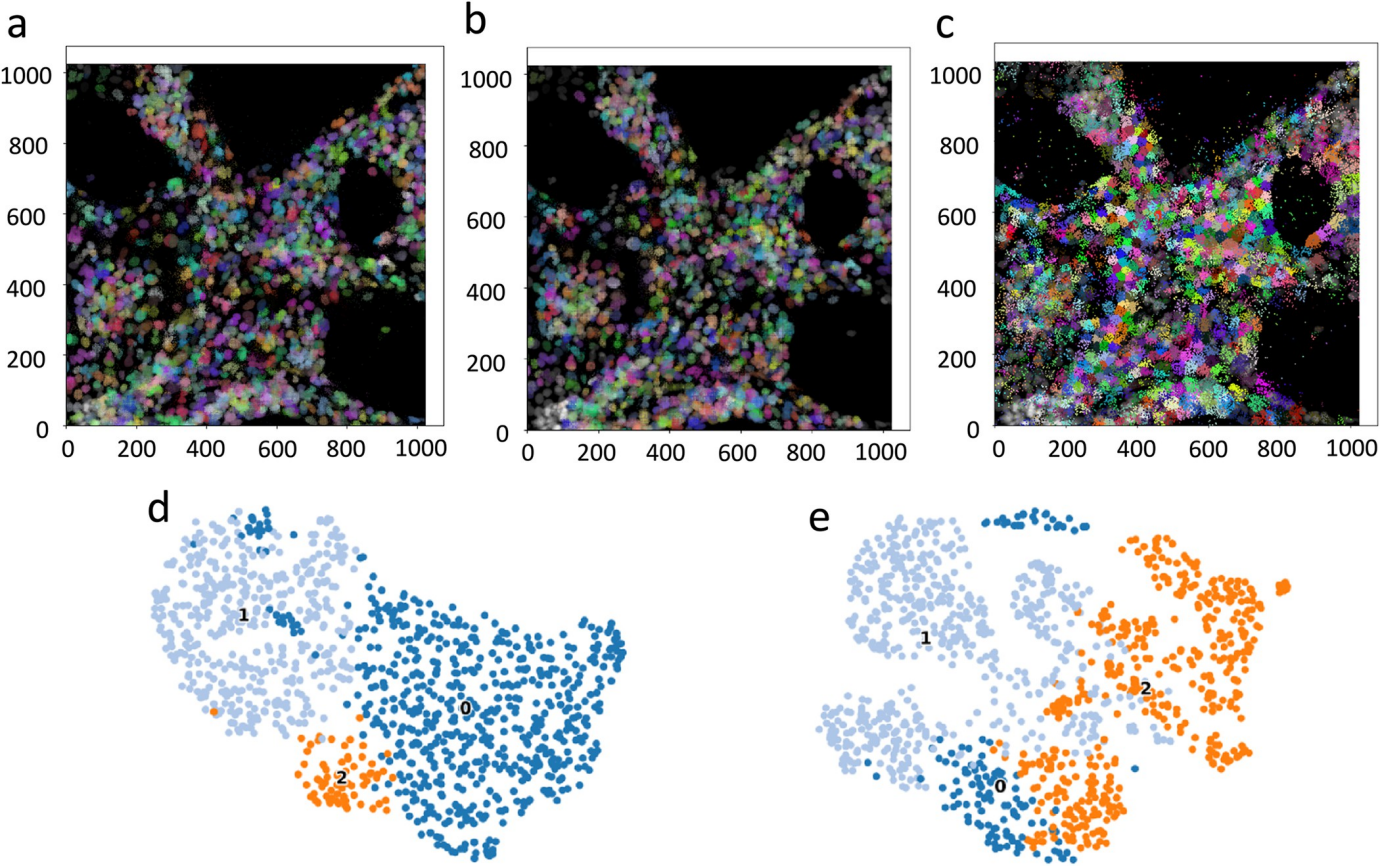
Visualization for the ground truth and cell segmentation performance of ST-CellSeg and ClusterMap on STARmap 8-gene data. (a) ground truth. (b) -(c) Cell segmentation results of ST-CellSeg and ClusterMap on the STARmap 8-gene dataset, respectively. Each color represents a cluster obtained through the clustering segmentation of cells. (d)-(e) Results of the data dimension reduction after applying ST-CellSeg and ClusterMap segmentation. Each color represents a specific cell type.

**Fig 3** illustrates the visualization of the ground truth, cell segmentation and classification results for ST-CellSeg and ClusterMap. The reason for choosing ClusterMap as a comparison is that the clustering analysis of this method on various datasets is the best among the six selected baseline methods, as shown in **Table 3**. Comparing Panel (a)–the ground truth of the STARmap 8-gene dataset, the cell segmentation results of the proposed method ST-CellSeg (Panel (b)), which focuses on multiscale information of local manifolds, are more compact in comparison to those of ClusterMap (Panel (c))). The biological significance of the segmented cells can be visually verified by the dimension reduction of the gene types and numbers contained in each cell. Thus, if the data dimension reduction results are compact, the segmented cells can be considered to have biological significance. Panels (d) and (e) are the results of the data dimension reduction after applying ST-CellSeg and ClusterMap segmentation, respectively. The results of ST-CellSeg are more compact and the segmented cells are considered to be biologically meaningful. The visualization results demonstrate that our method has a better performance on the cell segmentation task compared to the selected baseline methods. The segmentation visualization results of the other five baselines can be found in **S1 Table**.

### 3.2. Ablation studies

The innovation of our method lies in the sensing range of multiple scales. To verify the effectiveness of our innovation, we conduct a series of ablation experiments to compare the performance of the single-scale and multi-scale versions of the method. The experimental results are shown in **Table 4**. Overall, the multi-scale method performs better than the single-scale method. With the increasing R, the performance becomes relative better for all the three data sets.

**Table 4:** Ablation analysis of ST-CellSeg in terms of cluster methods and three different levels of sensing scales.

Datasets	STARmap 903-gene			STARmap 8-gene			MERFISH 140-gene		
Methods/Metric	ARI	NMI	SC	ARI	NMI	SC	ARI	NMI	SC
DBSCAN (R, 3R, 5R)	0.84	0.96	0.86	0.85	0.95	0.90	0.81	0.91	0.91
DBSCAN (R, 3R)	0.82	0.93	0.85	0.83	0.93	0.87	0.79	0.90	0.91
DBSCAN (R)	0.79	0.90	0.85	0.83	0.91	0.85	0.79	0.89	0.90

### 3.3. Time complexity of model computation

To evaluate the computational efficiency of ST-CellSeg in comparison to baseline methods, we calculate the floating-point operations per second (FLOPs) and the actual time of each method when applying to STARmap 903-gene, STARmap 8-gene, and MERFISH 140-gene data. The results reported in **Table 5** show that ST-CellSeg has lowest FLOPs in each dataset. In terms of the actual computational time, as shown in the **S2 Table**, ST-CellSeg has smaller computational time than ClusterMap on CPU-based machine. Although it has comparable computational time to the other 5 baselines, they work on the GPU-based machine. Thus, the proposed method is overall more efficient than the other six baselines in cell segmentation.

**Table 5:** Floating point operations per second (FLOPs) of ST-CellSeg and five baseline models on the three different datasets.

Datasets	ST-CellSeg	ClusterMap	SpaGCN	STAGATE	Cellpose	StarDist
STARmap 903-gene data	6.36M	7.78M	8.37M	6.93M	8.64M	8.98M
STARmap 8-gene data	11.59M	18.35M	23.45M	19.54M	19.31M	18.21M
MERFISH 140-gene data	16.87M	18.35M	18.63M	17.65M	23.65M	24.25M

## 4. Conclusion and discussion

We present a method called ST-CellSeg for cell segmentation tasks of spatial transcriptomics. ST-CellSeg is a manifold learning method that uses local multiscale information. We validate ST-CellSeg on three datasets including STARmap 903-gene and compare its performance to six baseline methods. The cell segmentation results show that the proposed method outperforms other algorithms on three clustering metrics ARI, NMI, and SC. The results of visual cell segmentation show that the cells obtained by ST-CellSeg segmentation are more compact and have more biological significance. Moreover, the results of ablation experiments show that the introduction of local multiscale information helps to improve the performance of cell segmentation, which proves the effectiveness of ST-CellSeg. In addition, we count the time complexity of different methods, and ST-CellSeg is overall faster than the comparison methods. Furthermore, as shown in S3 Table, the proposed method can handle the data sets with different cell shapes, use spatial information for cell segmentation and does not require GPU acceleration for data analysis. Hence, the ST-CellSeg is not only more accurate in segmentation results, but also efficient in computation.

## Supporting information

S1 Table The segmentation results of various methods for a more detailed comparison. (PDF)

S2 Table Comparison of cell segmentation times for different methods on different spatial transcriptomics datasets. In this supplementary material section, we provide additional details on the runtime comparison of cell segmentation methods using different computational platforms. The table presented below showcases the runtime performance of ST-CellSeg (CPU), ClusterMap (CPU), SpaGCN (GPU), STAGATE (GPU), Baysor (CPU), Cellpose (GPU), and StarDist (GPU) on two spatial transcriptomics datasets: STARmap 903-gene data and MERFISH 140-gene data.
(PDF)

S3 Table Comparison of cell segmentation methods based on different features and techniques. The table presents a comparison of various cell segmentation methods based on different features and techniques. ST-CellSeg, ClusterMap, SpaGCN, STAGATE, Baysor, Cellpose, and StarDist are evaluated in terms of their ability to handle cell shape, incorporate spatial information, operate without requiring labels, utilize deep learning techniques, and support GPU acceleration.
(PDF)
