# Supplementary material for: ST-CellSeg: Cell segmentation for imaging-based spatial transcriptomics using multi-scale manifold learning
Source: PLoS Comput Biol. 2024 Jun 27;20(6):e1012254. doi: 10.1371/journal.pcbi.1012254 (PMC11236102; doi:10.1371/journal.pcbi.1012254)
Supplement: S1 Table — (PDF) [file pcbi.1012254.s001.pdf]

S1 Table. The segmentation results of various methods for a more detailed comparison

We have conducted a comprehensive evaluation of ST-CellSeg alongside other existing cell segmentation methods. In this supplementary material, we present additional figures that showcase the segmentation results of various methods for a more detailed comparison.

	Segmentation Results
SpaGCN	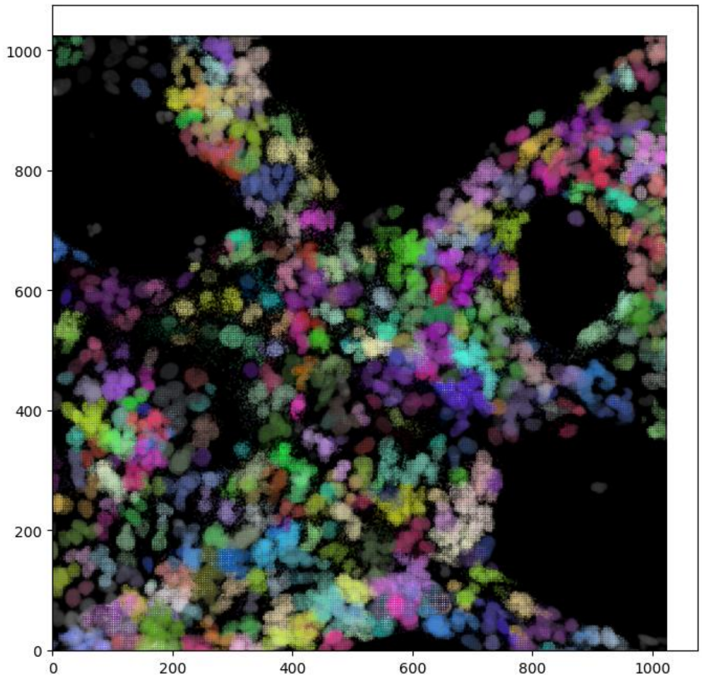

STAGATE	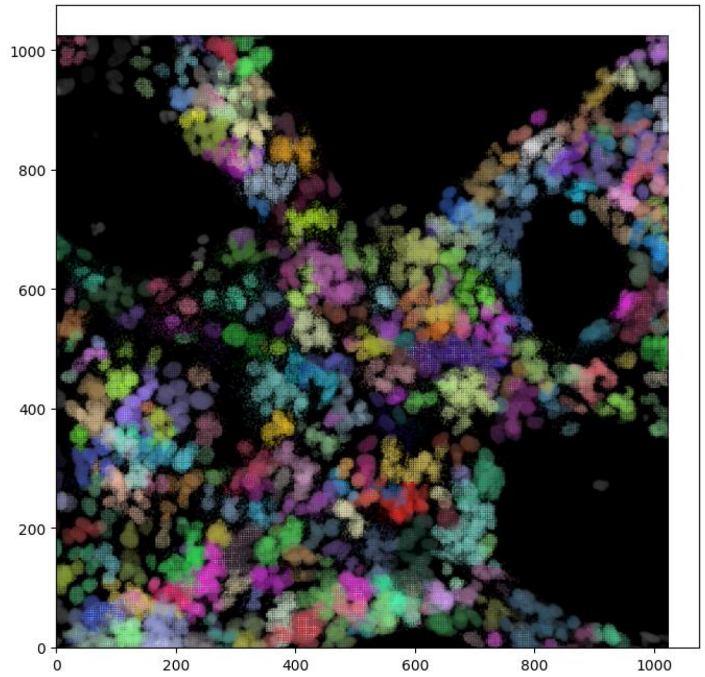
Baysor	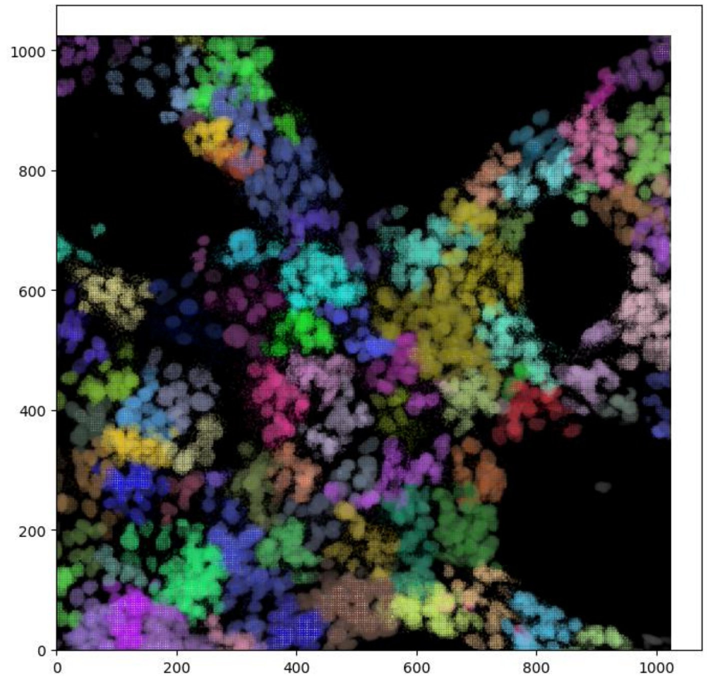

Cellpose	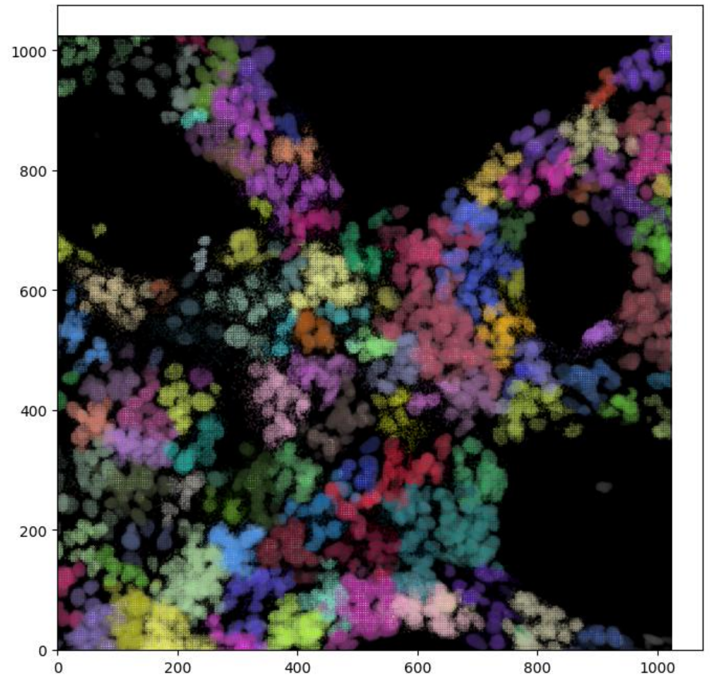
StarDist	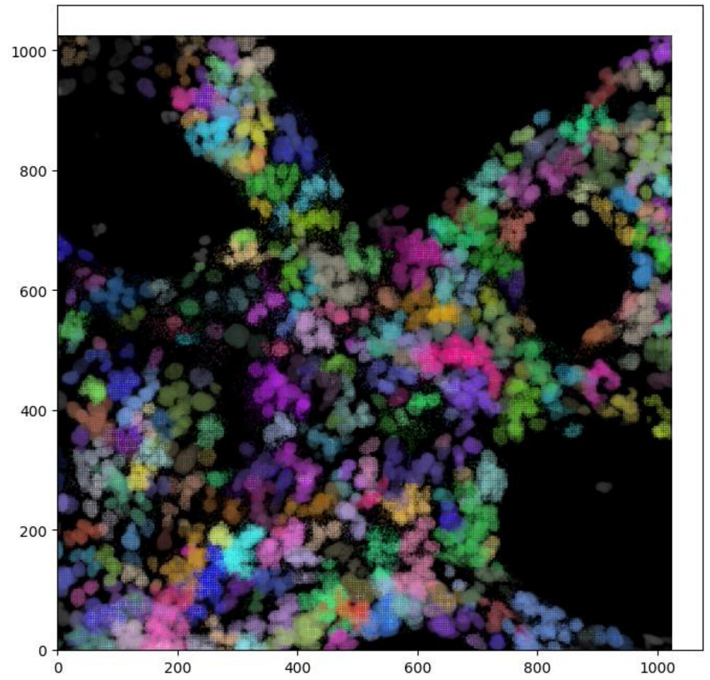
