# Supplementary material for: ST-CellSeg: Cell segmentation for imaging-based spatial transcriptomics using multi-scale manifold learning
Source: PLoS Comput Biol. 2024 Jun 27;20(6):e1012254. doi: 10.1371/journal.pcbi.1012254 (PMC11236102; doi:10.1371/journal.pcbi.1012254)
Supplement: S2 Table — In this supplementary material section, we provide additional details on the runtime comparison of cell segmentation methods using different computational platforms. The table presented below showcases the runtime performance of ST-CellSeg (CPU), ClusterMap (CPU), SpaGCN (GPU), STAGATE (GPU), Baysor (CPU), Cellpose (GPU), and StarDist (GPU) on two spatial transcriptomics datasets: STARmap 903-gene data and MERFISH 140-gene data. (PDF) [file pcbi.1012254.s002.pdf]

S2 Table. Comparison of cell segmentation times for different methods on different spatial transcriptomics datasets

In this supplementary material section, we provide additional details on the runtime comparison of cell segmentation methods using different computational platforms. The table presented below showcases the runtime performance of ST-CellSeg (CPU), ClusterMap (CPU), SpaGCN (GPU), STAGATE (GPU), Baysor (CPU), Cellpose (GPU), and StarDist (GPU) on two spatial transcriptomics datasets: STARmap 903-gene data and MERFISH 140-gene data.

	ST-CellSeg (CPU)	ClusterMap (CPU)	SpaGCN (GPU)	STAGATE (GPU)	Baysor (CPU)	Cellpose (GPU)	StarDist (GPU)
STARmap 903-gene data	3min 5s	3min 42s	2min 27s	2min 39s	4min 1s	3min 7s	3min 21s
STARmap 8-gene data	284min 28s	324min 48s	212min 31s	225min 16s	438min 8s	290min 54s	256min 48s
MERFISH 140-gene data	195min 2s	212min 38s	129min 49s	135min 19s	312min 17s	180min 19s	194min 31s
