# Supplementary material for: ST-CellSeg: Cell segmentation for imaging-based spatial transcriptomics using multi-scale manifold learning
Source: PLoS Comput Biol. 2024 Jun 27;20(6):e1012254. doi: 10.1371/journal.pcbi.1012254 (PMC11236102; doi:10.1371/journal.pcbi.1012254)
Supplement: S3 Table — The table presents a comparison of various cell segmentation methods based on different features and techniques. ST-CellSeg, ClusterMap, SpaGCN, STAGATE, Baysor, Cellpose, and StarDist are evaluated in terms of their ability to handle cell shape, incorporate spatial information, operate without requiring labels, utilize deep learning techniques, and support GPU acceleration. (PDF) [file pcbi.1012254.s003.pdf]

S3 Table. Comparison of cell segmentation methods based on different features and techniques

The table presents a comparison of various cell segmentation methods based on different features and techniques. ST-CellSeg, ClusterMap, SpaGCN, STAGATE, Baysor, Cellpose, and StarDist are evaluated in terms of their ability to handle cell shape, incorporate spatial information, operate without requiring labels, utilize deep learning techniques, and support GPU acceleration.

ST-CellSeg demonstrates strengths in capturing cell shape and spatial information, making it suitable for cell segmentation tasks. It also operates without the need for labeled data but does not utilize deep learning techniques or GPU acceleration. ClusterMap, on the other hand, excels in exploiting cell shape but lacks spatial information handling capabilities. It also does not rely on deep learning or GPU acceleration. SpaGCN and STAGATE emphasize the utilization of spatial information, making them well-suited for spatial cell segmentation tasks. Both methods leverage deep learning techniques and support GPU acceleration for improved efficiency. Baysor shares similarities with ST-CellSeg and ClusterMap, focusing on cell shape but lacking spatial information handling capabilities. It also does not utilize deep learning or GPU acceleration. Cellpose and StarDist prioritize spatial information and incorporate deep learning techniques, making them versatile for various cell segmentation tasks. Both methods are GPU accelerated for enhanced performance.

	Cell Shape	Spatial Information	No labels required	Deep Learning	GPU Acceleration
ST-CellSeg	√	√	√	×	×
ClusterMap	√	×	√	×	×
SpaGCN	×	√	×	√	√
STAGATE	×	√	×	√	√

Baysor	√	×	√	×	×
Cellpose	×	√	×	√	√
StarDist	×	√	×	√	√
